# Inflammation and nutritional status indicators as prognostic indicators for patients with locally advanced gastrointestinal stromal tumors treated with neoadjuvant imatinib

**DOI:** 10.1186/s12876-023-02658-x

**Published:** 2023-01-23

**Authors:** Ping’an Ding, Jiaxiang Wu, Haotian Wu, Chenyu Sun, Honghai Guo, Scott Lowe, Peigang Yang, Yuan Tian, Yang Liu, Lingjiao Meng, Qun Zhao

**Affiliations:** 1 The Third Department of Surgery, The Fourth Hospital of Hebei Medical University, Shijiazhuang, 050011 Hebei China; 2 Hebei Key Laboratory of Precision Diagnosis and Comprehensive Treatment of Gastric Cancer, Shijiazhuang, 050011 China; 3 Research Center of the Fourth Hospital of Hebei Medical University, Shijiazhuang, 050011 China; 4 AMITA Health Saint Joseph Hospital Chicago, 2900 N. Lake Shore Drive, Chicago, IL 60657 USA; 5 College of Osteopathic Medicine, Kansas City University, 1750 Independence Ave, Kansas City, MO 64106 USA

**Keywords:** Inflammation, Prognostic nutritional index, Neoadjuvant therapy, Gastrointestinal stromal tumors

## Abstract

**Background:**

Previous studies have confirmed that preoperative nutritional-inflammatory indicators can predict prognosis in various malignancies. However, to the best of our knowledge, no study has investigated the assessment of systemic inflammatory immunity index (SII) combined with prognostic nutritional index (PNI) scores to predict prognosis after neoadjuvant treatment with imatinib in locally advanced gastrointestinal stromal tumours (LA-GIST). The aim of this study was to evaluate the predictive value of pretreatment SII-PNI scores in predicting recurrence after neoadjuvant therapy with imatinib in patients with LA-GIST.

**Methods:**

We retrospectively analyzed 57 patients with LA-GIST who received imatinib neoadjuvant from January 2013 to March 2019. Patients were divided into recurrence and non-recurrence groups according to their follow-up status, and SII and PNI cut-offs were calculated by receiver operating characteristic. The SII-PNI score ranged from 0 to 2 and were categorized into the following: score of 2, high SII (≥ 544.6) and low PNI (≤ 47.2); score of 1, either high SII (≥ 544.6) or low PNI (≤ 47.2); score of 0, no high SII (≥ 544.6) nor low PNI (≤ 47.2).

**Results:**

All patients received imatinib neoadjuvant therapy for a median treatment period of 8.5 months (ranging from 3.2 to 12.6 months), with 8 patients (14.04%) and 49 patients (85.96%) developing recurrence and non-recurrence, respectively. Patients with a high SII-PNI score had a significantly worse recurrence-free survival time than those with a low SII-PNI score (*P* = 0.022, 0.046), and had a poorer pathological response (*P* = 0.014). Multivariate analysis demonstrated that the SII-PNI score was an independent prognostic factor for prediction of recurrence-free survival (*P* = 0.002).

**Conclusion:**

The pre-treatment SII-PNI score can be used to predict the efficacy after neoadjuvant treatment with imatinib in patients with LA-GIST, which may be a promising predictor of recurrence-free survival time for patients.

## Introduction

Gastrointestinal stromal tumours (GIST) are the most common mesenchymal derived tumours and most patients have mutations in the c-kit or platelet derived growth factor receptor alpha (PDGFRA) gene [1, 2]. Currently, the preferred treatment for GIST patients is surgical resection, with the goals of obtaining R0 margins, avoiding intraoperative tumour rupture and maximising preservation of organ function [3, 4]. Nevertheless, a growing number of studies have found that direct surgical resection is difficult for patients with specific sites (oesophagogastric junction, low rectum, duodenum) and large tumour diameters [5, 6]. Recently, imatinib, a small molecule tyrosine kinase inhibitor (TKI), has been widely used as an adjuvant therapy for GIST patients after surgery as it can inhibit cell growth and promote apoptosis by blocking tyrosine kinase [7, 8]. Several retrospective studies have found that neoadjuvant treatment with preoperative imatinib can be attempted in patients with locally advanced GIST (LA-GIST) [9–11]. Meanwhile, a prospective study of 63 patients with LA-GIST (RTOG 0132) found that surgical resection after 8–12 weeks of preoperative neoadjuvant imatinib (600 mg/d) resulted in an R0 resection rate of 77% and a 5-years overall survival rate of 77% [12]. In addition, several multicentre prospective studies have shown the same results, suggesting that preoperative neoadjuvant therapy with imatinib is effective in the treatment of LA-GIST and has a good “down-staging” effect, improving the R0 resection rate, safety of surgery and facilitating the preservation of organ function [13, 14].

Unfortunately, not all patients benefit from those treatments, and some of them develop recurrent metastases within a short period of time after treatment. Currently, the risk of recurrence after neoadjuvant therapy for LA-GIST is assessed by the National Institutes of Health (NIH) (2008 modified version), the Armed Forces Institute of Pathology (AFIP) criteria, primary tumour site, tumour diameter and mitotic index [15]. However, these assessment indicators are considered only from the perspective of the original GIST and ignore the impact of the patient's inflammatory and nutritional status on the tumour during neoadjuvant therapy.

Presently, inflammation plays an important role in the pathogenesis of malignant tumors, and the latest view is that tumor-related inflammation is considered to be the seventh characteristic of tumors [16, 17]. Numerous studies have found that the systemic inflammatory response can disrupt the host's immune response and promote tumour cells to escape from immune surveillance, which further promotes the development of angiogenesis, invasion and metastasis [18, 19]. At the same time, previous studies have found that the nutritional status of the tumour patient is one of the key factors influencing the progression of the tumour [20, 21]. The systemic immune-inflammatory index (SII) is a comprehensive measure of the body's systemic inflammatory and immune status consisting of peripheral blood neutrophils, lymphocytes and platelets [22]. Accumulating evidence has revealed that SII is closely related to the prognosis of various malignant tumors [22–24].

Our previous study also found that 10.09% of newly diagnosed GIST patients had malnutrition, and most of them were middle- and high-risk types [25]. Further follow-up found that the nutritional status of patients was also closely related to prognosis. Therefore, we hypothesise that poor nutritional status during neoadjuvant therapy in patients with LA-GIST may also be strongly associated with poor prognosis. The prognostic nutritional index (PNI), as a simple and feasible nutritional assay, formed based on the combination of lymphocyte count and albumin levels, has been shown to be associated with the prognosis of various malignancies and is widely used to assess the prediction of the efficacy of neoadjuvant therapy and the assessment of prognosis in cancer patients [26, 27].

In previous studies we have found that a new scoring system combining SII with PNI in immunotherapy for locally progressive gastric cancer [28] and in conversion therapy for advanced gastric cancer [29] has good diagnostic value in predicting the efficacy and assessing prognosis. However, previous studies have generally used a single haematological index, including SII [30] and PNI [31], and few studies have used SII in combination with PNI to assess the evaluation of efficacy and prediction of prognosis in LA-GIST patients after neoadjuvant therapy with imatinib. Therefore, in this study, we evaluated the predictive value of the pre-treatment SII-PNI score on the efficacy and prognosis of patients with LA-GIST receiving neoadjuvant therapy with imatinib.

## Materials and methods

### Study design and participants

This study retrospectively analyzed 57 patients with LA-GIST who underwent neoadjuvant imatinib therapy in the Fourth Hospital of Hebei Medical University from January 2013 to March 2019. The following inclusion criteria were applied: (1) all patients had histopathologically confirmed GIST; (2) genetic tests suggested the imatinib treatment was indicated; (3) age between 18 and 75 years; (4) preoperative imaging examination showed that the lesions were locally advanced, and surgery without pre-operation chemotherapy or radiation therapy may have a significant impact on the quality of life, including: the tumor site ≤ 5 cm from the cardia, ≤ 5 cm from anal dentate line, ≤ 5 cm from duodenal papilla, pancreaticoduodenectomy or combined organ resection is required for surgery; tumor diameter ≥ 10 cm; (5) all patients were treated with radical surgery after neoadjuvant treatment with imatinib; (6) complete hospitalization data, including computed tomography (CT) scans and follow-up data before and after neoadjuvant treatment, were available. Patients were excluded if they presented with the following: (1) the presence of concurrent tumors other than LA-GIST; (2) the presence of acute bleeding, perforation, and obstruction requiring emergency surgery; (3) poor functional reserve of organs that cannot tolerate surgery or patient refusal to undergo surgical treatment, or patient inability to cooperate with treatment; (4) the presence of lumbar spine metal implants; (5) concurrent history of other tumours or haematological disorders and (6) pre-operative co-infection and abnormal blood results. This study was tested and approved by the ethics committee of the Fourth Hospital of Hebei Medical University. All patients provided informed consent.

### Imatinib neoadjuvant therapy

The decision to administer imatinib neoadjuvant therapy was made by a multidisciplinary panel of surgeons, oncologists, pathologists, and radiologists after all patients were diagnosed with LA-GIST. The initial dose of imatinib was determined based on the results of genetic testing, which resulted in a dose of 400 mg/d for the KIT exon 11 mutation.

According to the National Comprehensive Cancer Network (NCCN) and the Chinese Society of Clinical Oncology (CSCO) guidelines for the treatment of GIST, the recommended duration of pre-operative neoadjuvant imatinib treatment is 6–12 months to maximize the effectiveness of the drug [32, 33]. The optimal timing of surgery was chosen if the either of the two following criteria was met: (1) two consecutive CT scans revealed no regression of the tumor; (2) surgery was considered by the surgeon to be radical and/or organ-preserving. All patients were treated surgically after 1 week of discontinuation of imatinib.

### Assessments

During neoadjuvant treatment, abdominal CT examination was performed every 3 months during preoperative treatment and the efficacy was assessed according to the Choi criteria [34]. A complete response (CR) is defined as the disappearance of all lesions and no new lesions after neoadjuvant therapy. In contrast, a partial response (PR) was defined as ≥ 10% reduction in tumour length and/or ≥ 15% reduction in tumour density, with no new lesions and no significant progression of non-measurable lesions. Progressive disease (PD) is defined as an increase in tumour length of ≥ 10% and tumour density that does not meet the criteria for PR, or the presence of a new lesion, or a new intratumoural nodule, or an increase in the size of an existing intratumoural nodule. In contrast, those that do not meet the criteria for CR, PR and PD are defined as stable disease (SD).

The criteria for evaluating the pathological efficacy of LA-GIST patients after neoadjuvant therapy were based on the “Chinese consensus guidelines for diagnosis and management of gastrointestinal stromal tumor” published in 2017 [35], which classified postoperative pathological specimens into mild effect (≤ 10%), low effect (> 10% and < 50%), moderate effect (≥ 50% and ≤ 90%), and high effect (> 90%) according to the percentage of necrotic degeneration areas in the tumor tissues. In this study, the mild and low effects were combined into a low response group, and the moderate and high effects were combined into a high response group.

### Definitions and follow-up

Peripheral venous blood samples were collected in fasting state within 1 week before initiation of chemotherapy in all patients. Peripheral neutrophil, lymphocyte and platelet counts were measured and analysed using an automated haematology analyser (Beckman Coulter LH750) and albumin levels were measured and analysed using an automated haematology analyser (Beckman Coulter AU5800), respectively. Referring to the results in our previous studies, in this study PNI was defined as PNI = albumin (g/L) + 5 × total lymphocyte count (10^9^/L) and SII was defined as SII = platelets × neutrophil/lymphocyte count [28, 29].

All patients were recommended to have an enhanced CT scan of the abdomen every 3 months for the first 3 years postoperatively and every 6 months for the 4–5th years. Follow-up methods mainly included telephone encounter, outpatient visits, and hospitalization. In this study, our primary observational endpoint was recurrence-free survival (RFS), defined as the time from the start of neoadjuvant therapy to the date of documented relapse or death from any cause at follow-up, and the follow-up deadline date for this study was January 31, 2022.

### Statistical analyses

SPSS version 26.0 and GraphPad Prism 8.01 were utilized to perform statistical analyses. The optimal cut-off values for SII and PNI with the highest Youden index were determined by plotting the receiveroperator characteristic curve (ROC) based on the patient's RFS survival time. Survival analysis was performed using the Kaplan–Meier method. The change values of tumor diameter measured by CT before and after neoadjuvant therapy in LA-GIST patients were plotted by GraphPad Prism 8.01 software to assess the waterfall of CT imaging efficacy for each patient. Univariate and multivariate analyses were investigated by the Cox proportional hazards regression model. The hazard ratio (HR) and 95% confidence interval (CI) were used to assess relative risks. Spearman correlation analysis was used to evaluate the relationship between PNI and SII. *P* values < 0.05 were considered as statistically significant.

## Results

### Patients’ demographic information and tumor characteristics

**Fig. 1:**
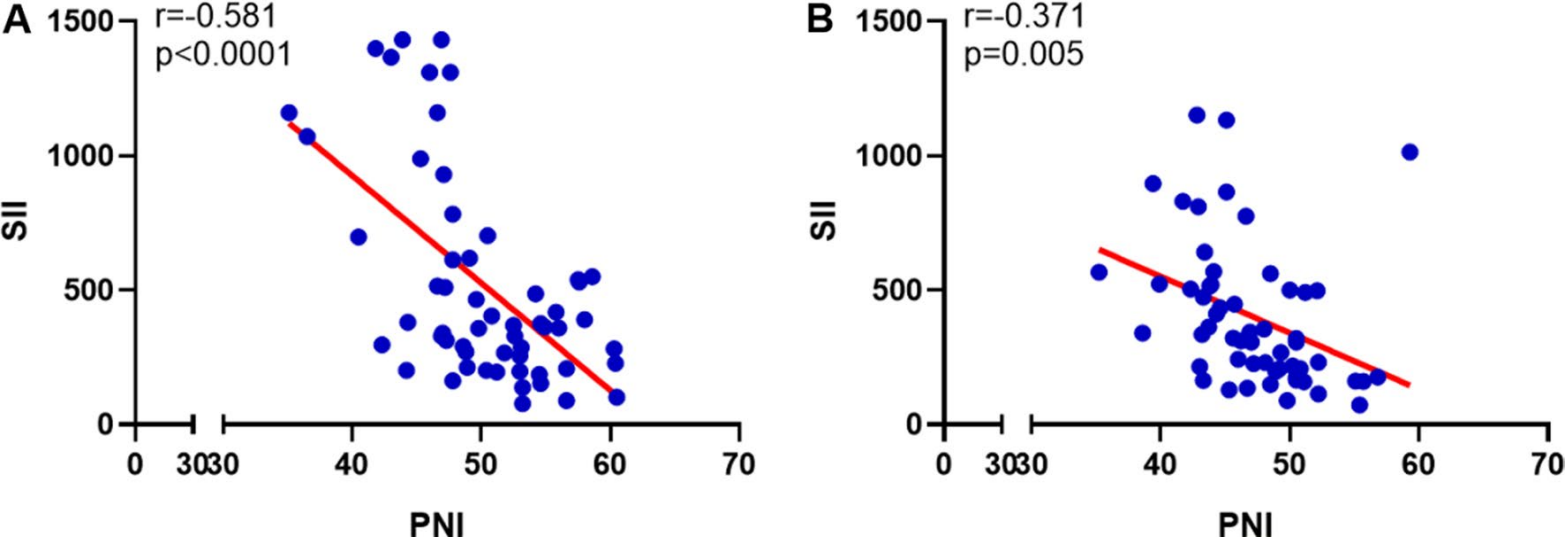
Correlation analysis between SII and PNI. **A** Pre-imatinib neoadjuvant therapy; **B** post-imatinib neoadjuvant therapy

According to the inclusion and exclusion criteria of this study, a total of 57 patients with LA-GIST were included, of whom 38 (66.67%) were male and 19 (33.33%) were female. Patient demographic information and pathological features are shown in Table 1. The mean age of the patients was 57.4 ± 10.7 years (ranging from 30 to 82 years old), of which 45.61% were ≥ 60 years. The mitotic numbers per HPF were 0–5 and ≥ 5 in 8 (14.04%) and 49 (85.96%) patients, respectively. The median SII and PNI before neoadjuvant therapy with imatinib were 369.7 (ranging from 77.5 to 1432.0) and 49.8 (ranging from 35.1 to 60.5), respectively, and there was a strong negative correlation between them (r = − 0.581, *P* < 0.0001; Fig. 1A). And after neoadjuvant treatment SII and PNI were 321.3 (ranging from 72.6 to 1152.2) and 46.6 (ranging from 35.2 to 55.7) respectively, which also had a moderate negative correlation (r = − 0.371, *P* = 0.005; Fig. 1B).

**Table 1:** Patient and tumor characteristics

Patient demographic information/tumor characteristics	Case (%)
*Sex*	
Male	38 (66.67)
Female	19 (33.33)
*Age (years)*	
< 60	31 (54.39)
≥ 60	26 (45.61)
*ECOG performance status*	
0	49 (85.96)
1	8 (14.04)
*Tumor size (cm)*	
< 10.0	18 (31.58)
≥ 10.0	39 (68.42)
*BMI (Kg/m*^*2*^*)*	
< 18.5	3 (5.26)
18.5–24.5	38 (66.67)
≥ 24.5	16 (28.07)
*Lesion site*	
Stomach	49 (85.96)
Duodenum	1 (1.75)
Mesentery	3 (5.26)
Colon	1 (1.75)
Rectum	3 (5.26)
*Mitosis (HPF)*	
0–5	8 (14.04)
≥ 5	49 (85.96)

### Optimal cut-off values of SII and PNI before and after neoadjuvant therapy

**Fig. 2:**
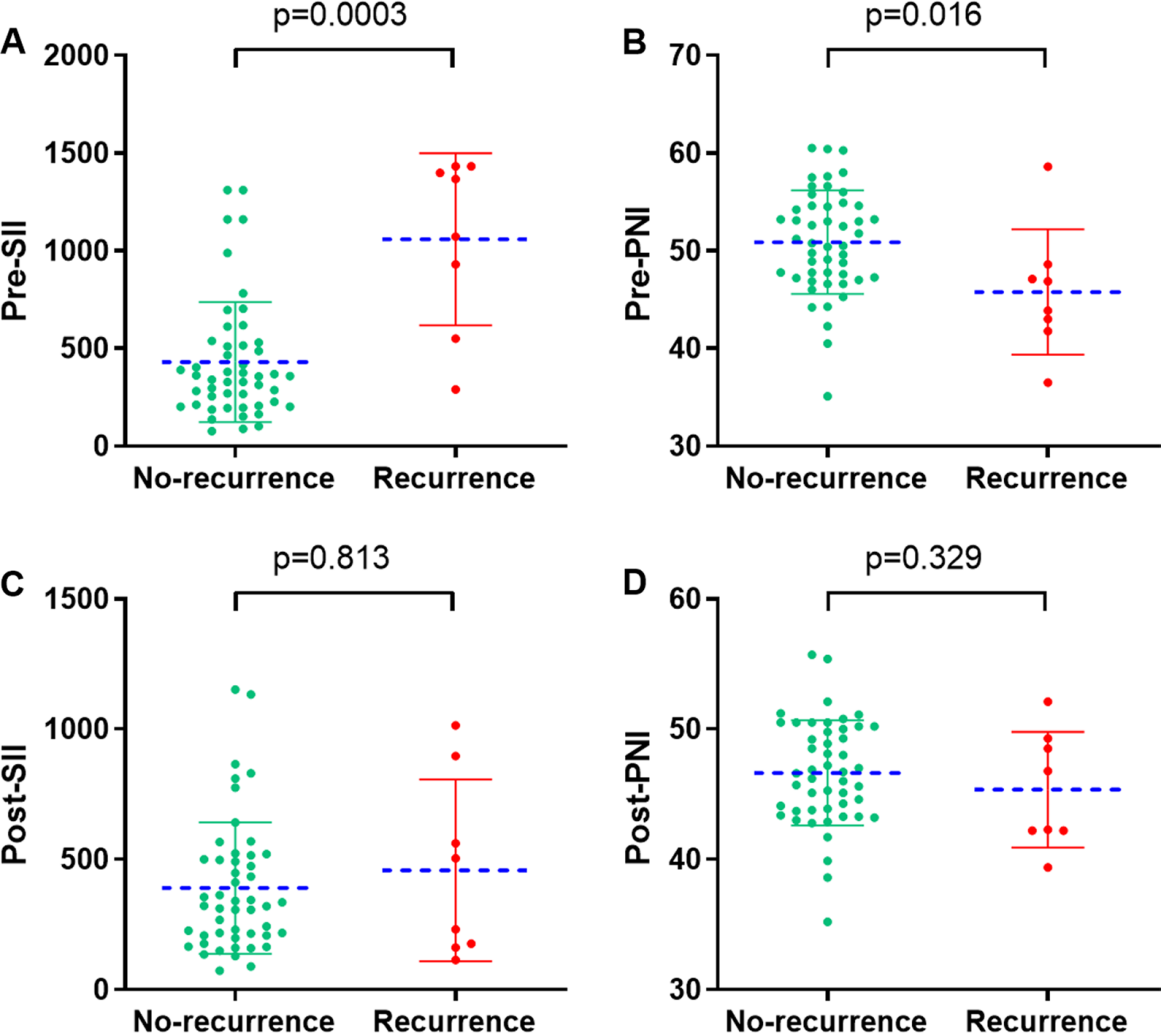
Relationship between recurrence and the SII(A/C)/PNI(B/D). **A**, **B** Before neoadjuvant treatment; **C**, **D** after neoadjuvant treatment

At the time of follow-up, eight patients in the group had recurrence, including five with liver metastases, two with abdominal metastases and one with anastomotic recurrence. The mean SII before neoadjuvant treatment was 431.6 ± 306.7 and PNI was 50.9 ± 5.3 in the 49 patients who did not relapse, while after treatment it was 390.0 ± 252.4 and 46.6 ± 4.0, respectively. In addition, for the eight patients who developed recurrence before neoadjuvant treatment the mean SII and PNI were 1059.0 ± 440.1 and 45.8 ± 6.4, respectively, while after treatment the mean SII was 457.9 ± 348.6 and PNI was 45.4 ± 4.4. We observed that SII was significantly higher in patients with recurrence before neoadjuvant treatment (*P* = 0.0003), but PNI was significantly lower in patients without recurrence (*P* = 0.016) (Fig. 2A, B). However, this difference between the two groups of patients was not as significant after neoadjuvant therapy for either SII (*P* = 0.813) or PNI (*P* = 0.329) (Fig. 2C, D).

**Fig. 3:**
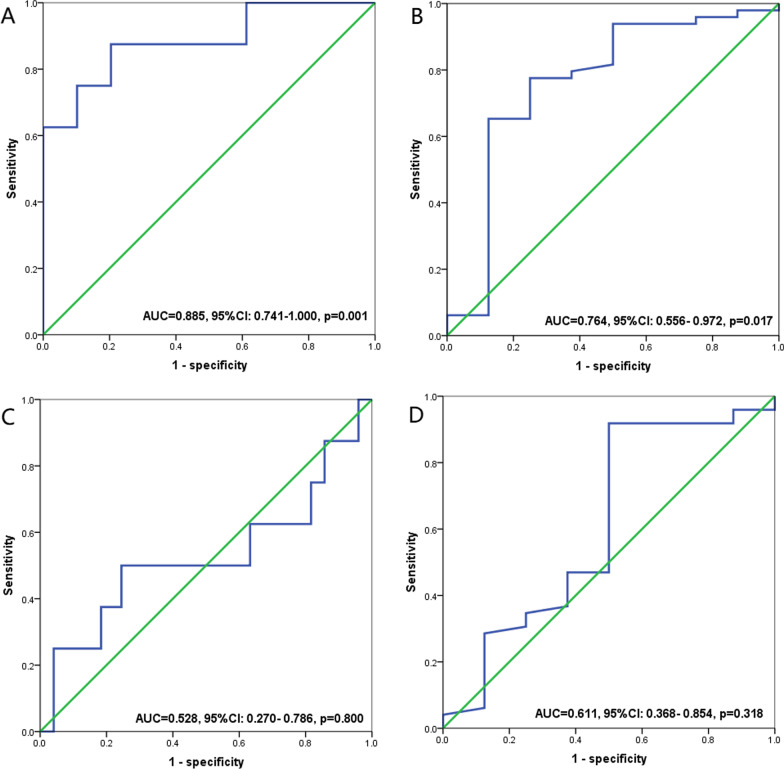
ROC curves for discriminating patients with recurrence and those with non-recurrence according to values of the SII (A/C) and PNI(B/D). **A**, **B** Before neoadjuvant treatment; **C**, **D** after neoadjuvant treatment

To determine the optimal cut-off values for the continuous variables of SII and PNI, we constructed ROC curves and calculated AUC to assess the predictive ability of SII and PNI in terms of differentiating between patients experiencing recurrence and non-recurrence before and after neoadjuvant treatment with imatinib. SII and PNI before neoadjuvant therapy had good discriminatory ability with optimal cut-off values of 544.6 (AUC = 0.885, 95% CI 0.741–1.000, *P* = 0.001; sensitivity of 0.875 and specificity of 0.796) and 47.2 (AUC = 0.764, 95% CI 0.556–0.972, *P* = 0.017; sensitivity of 0.776 and specificity of 0.750) (Fig. 3A, B). However, after neoadjuvant treatment SII (AUC = 0.528, 95% CI 0.270–0.786, *P* = 0.800) and PNI (AUC = 0.611, 95% CI 0.368–0.854, *P* = 0.318) failed to accurately distinguish between recurrent and non-recurrent patients (Fig. 3C, D). Therefore, all LA-GIST patients were divided into three groups based on the optimal cut-off values for SII and PNI before imatinib neoadjuvant therapy: score 2 (n = 11), high SII (≥ 544.6) and low PNI (≤ 47.2); score 1 (n = 13), high SII (≥ 544.6) or low PNI (≤ 47.2); and score 0 (n = 33), no high SII (≥ 544.6) or low PNI (≤ 47.2).

### The relationship between SII-PNI score and neoadjuvant therapy response

**Fig. 4:**
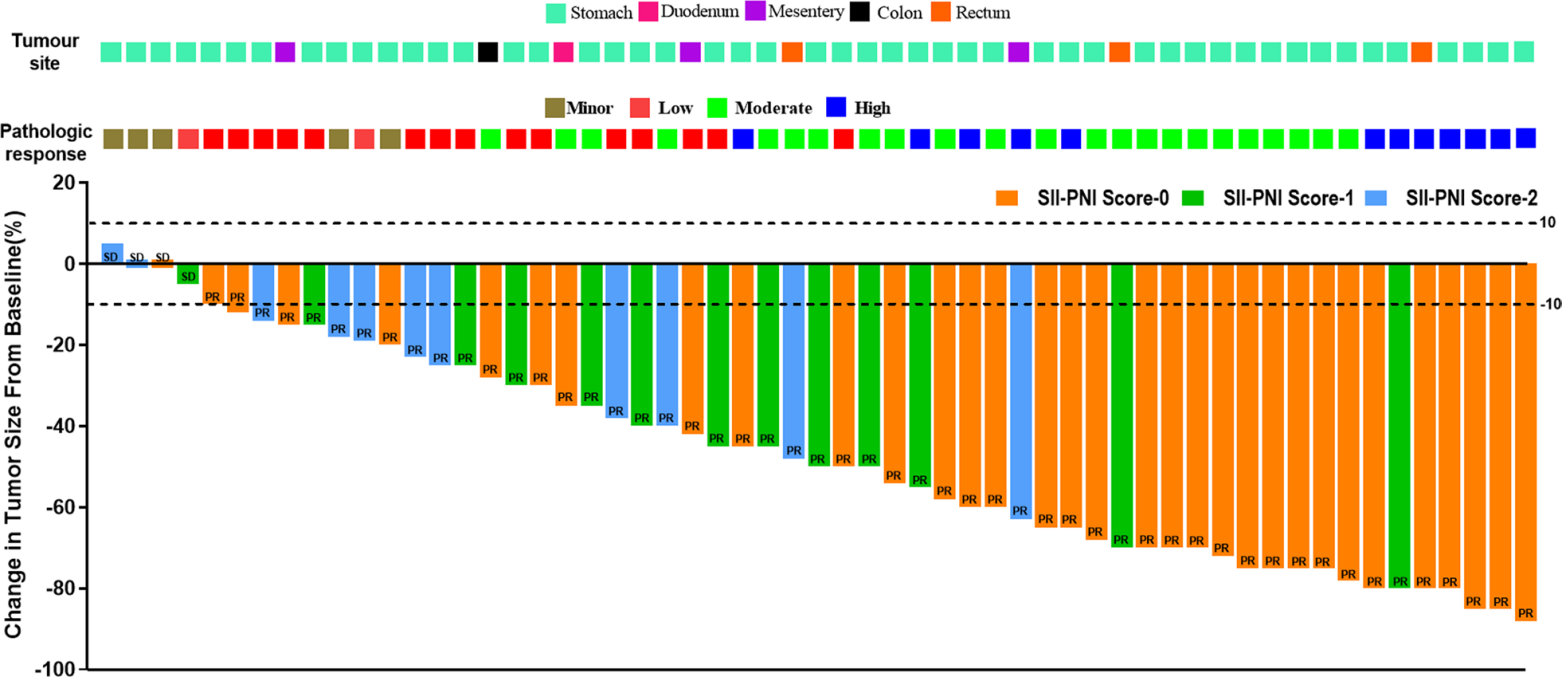
A waterfall plot of ranked best tumor shrinkage. Dashed lines indicate a 10% increase in tumor diameter from baseline for progression (progressive disease) and 10% for tumor regression (partial response). Of all LA-GIST patients treated with neoadjuvant imatinib, only 4 of 57 (7.02%) showed stable disease, while the remaining 53 patients (92.98%) showed partial responses and none showed disease progression

All patients received imatinib neoadjuvant therapy for a median treatment period of 8.5 months (ranging from 3.2 to 12.6 months) with no discontinuations during treatment. 57 patients with LA-GIST completed abdominal CT-enhanced scans before and after neoadjuvant therapy and only 4 patients (7.02%) had SD according to the Choi criteria, the remaining patients had PR (Fig. 4). There were no significant differences between the groups with different SII-PNI scores in the evaluation of imaging efficacy (*P* = 0.233), but changes in pathological response were significantly different between the groups (*P* = 0.014), with the lower the score, the more pronounced the changes (Table 2).

**Table 2:** Relationship between tumor response and the SII-PNI score

SII-PNI score	N	Imaging efficacy		Pathological response	
		SD	PR	Minor + low	Moderate + high
0	33	1 (3.03%)	32 (96.97%)	8 (24.24%)	25(75.76%)
1	13	1 (7.69%)	12 (92.31%)	6 (46.15%)	7(53.85%)
2	11	2 (18.18%)	9 (81.82%)	8 (72.73%)	3(27.27%)
p		0.233		0.014	

### Relationship between SII-PNI score and prognosis

**Fig. 5:**
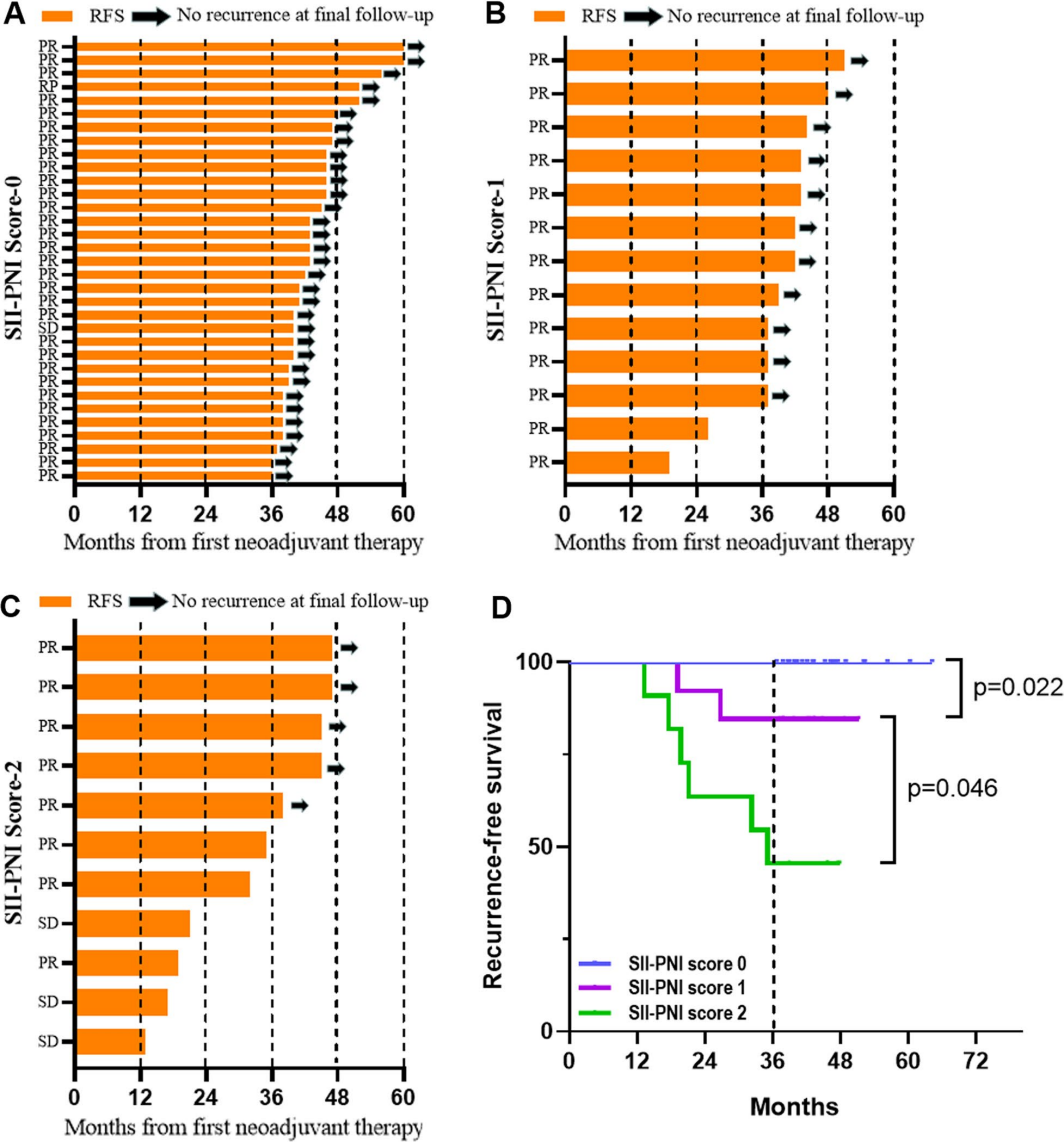
Recurrence-free survival of LA-GIST patients with different SII-PNI scores. **A** Recurrence-free survival in patients with SII-PNI score of 0; **B** recurrence-free survival in patients with SII-PNI score of 1; **C** recurrence-free survival in patients with SII-PNI score of 2; **D** comparison of non-recurrence survival time of patients with different SII-PNI scores

Fifty (87.72%) of the 57 patients with LA-GITS continued postoperative oral imatinib therapy at 400 mg/d, while the remaining 7 (12.28%) patients refused adjuvant therapy with postoperative imatinib. The median duration of oral imatinib in these 50 patients who received adjuvant therapy was 38.9 months (95% CI 15.3–62.4 months). All patients completed follow-up with a median follow-up period of 42.1 months (13.2–64.2 months), with no recurrent metastases in patients in the SII-PNI score group 0, and 2 and 6 recurrent metastases in patients in score groups 1 and 2, respectively. The 3-years recurrence-free survival (RFS) for the whole group was 85.96%, with the 3-years RFS for patients with a SII-PNI score of 0 being 100.00%, compared to 84.62% and 45.45% for patients with a score of 1 and 2 subgroups respectively, with significant differences between the three groups. The comparison of 3 years RFS among patients with different SII-PNI scores was statistically significant (all *P* < 0.05) (Fig. 5). Cox multivariate analysis showed that tumour response (*P* = 0.012), the SII-PNI score (*P* = 0.002), tumour size (*P* = 0.020) and postoperative imatinib treatment (*P* = 0.008) were independent risk factors for 3-years RFS (Table 3).

**Table 3:** Univariate and multivariate analyses of the clinicopathological characteristics for RFS

Independent factor	Univariate analysis			Multivariate analysis		
	Hazard ratio	95% CI	*P* value	Hazard ratio	95% CI	*P* value
*Sex*			0.682			
Female	1.000	Reference				
Male	1.324	0.512–1.761				
*Age (years)*			0.306			
< 60	1.000	Reference				
≥ 60	1.135	0.739–1.841				
*Imaging efficacy*			0.678			
PR	1.000	Reference				
SD	1.528	0.823–1.924				
*Tumor response*			0.004			0.012
Moderate/high	1.000	Reference		1.000	Reference	
Minor/low	5.412	2.421–11.898		3.341	2.832–7.081	
*SII-PNI score*			0.001			0.002
0	1.000	Reference		1.000	Reference	
1	2.679	1.276–5.822		2.127	1.231–4.245	
2	5.122	2.288–10.241		4.431	2.212–8.326	
*Tumor size (cm)*			0.002			0.020
< 10.0	1.000	Reference		1.000	Reference	
≥ 10.0	3.218	1.781–7.723		2.719	1.221–5.651	
*Lesion site*			0.581			
No-stomach	1.000	Reference				
Stomach	1.892	0.892–2.822				
*Postoperative imatinib treatment*			0.002			0.008
Yes	1.000	Reference		1.000	Reference	
No	2.256	1.466–3.564		3.561	1.754–7.791	

## Discussion

In recent years, with the rapid development of surgical techniques and multimodal therapies such as TKI molecular targeting drugs, the clinical outcomes and quality of life of GIST patients have improved significantly [3]. Nowadays, neoadjuvant treatment with imatinib for patients with LA-GIST has attracted much attention, with the following advantages [33, 35]: firstly, it can reduce the tumor volume and decrease the clinical stage; secondly, it can also reduce the scope of surgery and avoid unnecessary combined organ resection, reducing the risk of surgery and also increasing the chance of radical resection; in addition, it can protect the structure and function of important organs for tumors in specific sites; finally, for patients with large tumor diameter and high risk of intraoperative rupture, neoadjuvant treatment can reduce the possibility of drug-induced dissemination therapy. However, not all patients with LA-GIST benefit from this, with 10–20% of patients experiencing progression after 3 years of treatment [36]. In our cohort, we observed disease progression of 14.04% at 3 years after neoadjuvant imatinib treatment, which is consistent with previous studies [11, 36]. Currently, the common approach to predicting recurrence for LA-GIST is the 2008 revised NIH, AFIP and other guideline consensus, but these are only assessed from the perspective of postoperative pathology and do not take into account the dynamic changes in inflammatory and nutritional status of patients during neoadjuvant therapy [15].

Numerous studies have found that the biological behaviour of malignant tumourigenesis, development and invasion depends not only on the malignant characteristics of the tumour cells, but also on the tumour microenvironment [16, 18]. Inflammatory cells are considered to be an important component of the tumour microenvironment, and their mediated inflammatory response promotes invasion and metastasis by disrupting the immune response and further leading to immune escape of tumour cells [37]. Currently, a growing number of studies have found that inflammatory cells in peripheral blood can migrate through the body circulation to act in local tumour tissues, and therefore systemic inflammatory markers can be used to predict tumour prognosis in the tumour microenvironment in relation to the immune response [38]. The SII has received increasing attention as a more comprehensive measure of the status of the systemic inflammatory response, and several studies have confirmed its value in predicting tumour prognosis and outcome [18, 19, 23]. Besides, the nutritional status of GIST patients has received increasing attention in recent years. Our previous studies have demonstrated that nutritional status of GIST patients is a risk factor affecting the prognosis whether at the initial diagnosis or after surgery [22, 39]. The PNI is an index calculated from serum albumin and peripheral blood lymphocytes that provides a comprehensive reflection of the patient's nutritional status and immune function [26, 27]. To the best of knowledge, this is the first study to demonstrate the prognosis prediction of SII combined with PNI in patients with LA-GIST after neoadjuvant imatinib.

Our study showed that patients who developed recurrence had higher SII and lower PNI than the non-recurrence group at baseline, but this difference was less pronounced after neoadjuvant therapy. Meanwhile, we also found that the SII-PNI score correlated with pathological response after neoadjuvant chemotherapy, with the higher the score at baseline, the worse the pathological response in LA-GIST patients, which was consistent with our previous study [28, 29]. We speculate that the reasons for this outcome may include: firstly, elevated SII prior to neoadjuvant therapy indicates the presence of an inflammatory microenvironment that promotes tumor invasion and metastasis, whereas reduced PNI indicates poorer immune function and nutritional status, further promoting the formation of an inflammatory microenvironment as it was described by Yamanaka et al. [40]. Furthermore, epithelial-mesenchymal transition (EMT) mediated by the local inflammatory microenvironment of the tumor promotes tumor cell escape and resistance to therapeutic agents, which in turn affects the pathological response to neoadjuvant therapy in patients with LA-GIST [41, 42].

In recent years, there has also been increasing interest in the impact of SII and PNI on the prognosis of GIST patients. A retrospective study involving 431 GIST patients found that compared with the low PNI group (PNI < 47.45), the recurrence-free survival (RFS) of the high PNI group (PNI ≥ 47.45) was significantly prolonged (the 5-years RFS rates were 89.9% and 70.8%, respectively, *P* < 0.001) [31]. Interestingly, Elif Yuce et al. [43] found that PNI was not a risk factor for the prognosis of GIST patients. Furthermore, As Dolan et al. [44] published a retrospective analysis including 160 patients who underwent GIST surgery, the authors demonstrated that SII could be used as a prognostic predictor. Similar results were obtained in another retrospective analysis that included 45 GIST patients [30]. In this study, we also assessed the relationship between SII-PNI scores and neoadjuvant treatment recurrence in patients with LA-GIST. The 3-years RFS for patients with SII-PNI scores of 0 was 100.00% compared to 84.62% and 45.45% for patients with scores of 1 and 2 respectively, with significant differences between the three groups. The possible mechanism of SII-PNI predicting prognosis are as the followings: firstly, elevated neutrophils significantly inhibit lymphokine-activated killer cell-mediated cytotoxic effects, thereby down-regulating the patient's anti-tumor cell immune response and thus promoting tumor cell proliferation and migration [45, 46]; secondly, platelets promote tumour growth by secreting tumour growth factors such as platelet-derived growth factor (PDGF) and vascular endothelial growth factor (VEGF). Platelets can also play an integrative role in the process of tumor cell metastasis, causing tumor cells to evade the host immune system, thus protecting tumor cells from being easily recognized and facilitating tumor cell dissemination [47]; furthermore, lymphocytopenia suggests a decrease in the body's immune function, inducing apoptosis of tumor-specific T cells and inhibiting the activation and proliferation of T cells, further promoting the proliferation and migration of tumor cells [48, 49]; finally, systemic inflammatory responses exacerbate malnutrition and decreased body function in patients with malignancy, promoting poor prognosis in patients with malignancy [40].

It is noteworthy that a few limitations of current research also exist. Firstly, this study was a single-centre retrospective study with a relatively small number of patients included, which may have been subject to selection bias. Secondly, this study only analysed the relationship between SII-PNI scores and recurrence in LA-GIST patients, and not the overall survival time of patients. Therefore, more large-sample, prospective, multicentre studies are needed for validation in order to determine the predictive value of the parameters examined in the study.

## Conclusions

In conclusion, our study suggests that systemic inflammation and nutritional status are equally important in the tumourigenesis of LA-GIST cases. This study was the first to show that the SII-PNI score based on peripheral blood counts in LA-GIST patients is a promising predictor of pathological response and recurrence outcome after neoadjuvant therapy. These findings may facilitate the development of treatment strategies and clinical risk stratification.

## Data Availability

The datasets used and/or analyzed during the current study are available from the corresponding author on reasonable request.
